# Analysis of Temperature and Humidity Field in a New Bulk Tobacco Curing Barn Based on CFD

**DOI:** 10.3390/s17020279

**Published:** 2017-01-31

**Authors:** Zhipeng Bai, Duoduo Guo, Shoucang Li, Yaohua Hu

**Affiliations:** College of Mechanical and Electronic Engineering, Northwest A&F University, Yangling 712100, China; byson@nwsuaf.edu.cn (Z.B.); guoduoduo@nwsuaf.edu.cn (D.G.); 15591825515@163.com (S.L.)

**Keywords:** bulk tobacco curing barn, computational fluid dynamics, temperature and humidity field, sensors

## Abstract

A new structure bulk tobacco curing barn was presented. To study the temperature and humidity field in the new structure tobacco curing barn, a 3D transient computational fluid dynamics (CFD) model was developed using porous medium, species transport, κ-ε turbulence and discrete phase models. The CFD results demonstrated that (1) the temperature and relative humidity predictions were validated by the experimental results, and comparison of simulation results with experimental data showed a fairly close agreement; (2) the temperature of the bottom and inlet area was higher than the top and outlet area, and water vapor concentrated on the top and outlet area in the barn; (3) tobacco loading density and thickness of tobacco leaves had an explicit effect on the temperature distributions in the barn.

## 1. Introduction

In China, the bulk tobacco curing barn has been popularized during the last two decades due to high capacity and curing quality. In 1960, the first farm-scale operation in bulk curing was initiated in North Carolina [1]. Traditional natural ventilation used in the traditional tobacco curing barn was displaced by forced ventilation used in the bulk tobacco curing barn. Different from the traditional tobacco curing barn, the bulk tobacco curing barn added the hot air circulation system and the auto control system, which can save costs of energy and liberate manpower. Due to the application of forced ventilation technology, the diversity of structure of the bulk tobacco curing barn with different air flow directions is gaining a lot of interest in recent tobacco curing studies, specifically, the bulk tobacco curing barns with air rising [2,3], and air descending [4], which are the most common.

In general, tobacco flue curing can be divided into yellowing, leaf drying, and stem drying stages [5]. Changes in tobacco flue curing processes are mainly the result of water evaporation and enzymatic reaction [6]. In addition, the quality of curing tobacco is influenced by the air flows, temperature and relative humidity distribution in bulk tobacco curing barn.

Computational fluid dynamics (CFD) is increasingly being used to simulate flow field, mass and heat transfer. The capability of CFD modeling to predict air flows and temperature and relative humidity distributions for many applications in thermosyphon [7] and cool store [8] has been demonstrated. Temperature and relative humidity distributions in the tobacco drying process have been studied using artificial neural networks [9]. However, numerical studies focusing on tobacco curing barn were rarely considered. Zhang et al. [10] simulated the temperature field in tobacco oven during the drying process of cut tobacco based on the finite volume method. Wei et al. [11] studied the temperature distribution of tobacco curing barn with experiments and numerical simulation. Bao et al. [12] performed numerical studies for obtaining the temperature, humidity and velocity field in the tobacco curing barn. They applied a mixture model and porous medium to simulate the water evaporation process and tobacco leaves. Water-phase was introduced into the curing barn from inlet boundary. Tobacco leaves contained almost all the water in bulk tobacco curing barn. As the water came from tobacco leaves and evaporated in them, it is therefore necessary to develop a numerical model that would make possible to introduce water into the curing barn from tobacco leaves and predict temperature and relative humidity distributions accurately.

A new structure bulk tobacco curing barn heated by heat pump was presented and a 3D transient CFD model using the species transport and the discrete phase models to predict the temperature and humidity distributions in the bulk tobacco curing barn was developed in this study. The objectives of this study were (1) to validate the CFD model with experimental temperature and relative humidity data during the curing process; (2) to study the description of the temperature and water vapor mass fraction profiles in the tobacco curing barn; (3) to study the temperature distributions with different porosities and particle average diameters. This can be the basis for tobacco curing process and barn design optimization.

## 2. Materials and Methods

### 2.1. Description of the New Bulk Tobacco Curing Barn

**Figure 1:**
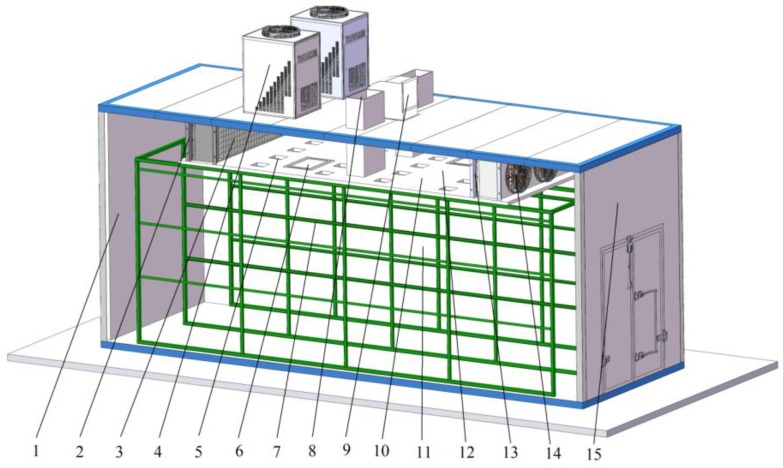
Structure of bulk tobacco curing barn. 1. Left wall; 2. Left fan unit; 3. Left radiator; 4. Heat pump; 5. Air holes; 6. Emergency gate; 7. Steel frame; 8. Moisture discharged holes; 9. Inlet; 10. Polyurethane foam sandwich panel; 11. Tobacco curing chamber; 12. Air return chamber; 13. Right radiator; 14. Right fan unit; 15. Right wall.

**Figure 2:**
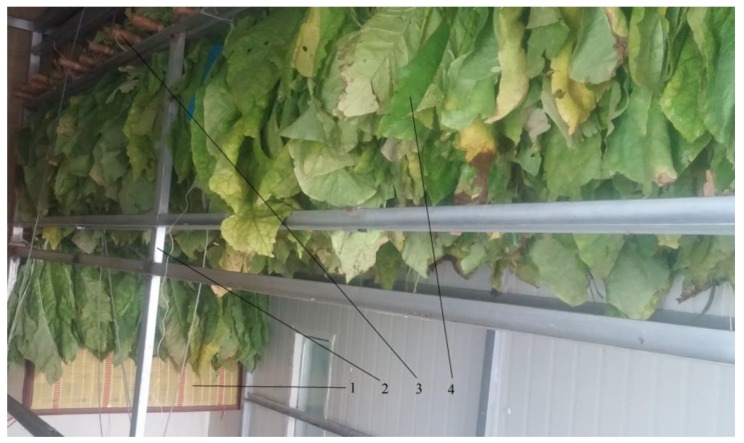
Tobacco curing chamber. 1. Bamboo curtain; 2. Steel frame; 3. Stick of bamboo; 4. Tobacco leaves.

The new structure bulk tobacco curing barn was 8.2 m long, 2.9 m wide and 3.6 m high, and its total volume was 85 m^3^. The barn contained two main parts: a tobacco curing chamber and an air return chamber. The ahead vertical wall was hidden to show internal structure in the curing barn as shown in Figure 1. The tobacco curing chamber and the air return chamber were separated by a 0.1 m thick layer of polyurethane foam sandwich panel, and two heat pumps on the roof provided heat for the radiators on the both left and right sides of the polyurethane foam sandwich panel. There were air holes and emergency gates on the polyurethane foam sandwich panel. A steel frame which was 7.3 m long, 2.7 m wide and 2.6 m high was placed in curing chamber. Tobacco leaves were tied along sticks of bamboo before being placed on the steel frame. And bamboo curtains were hung on both sides of steel frame to protect tobacco leaves as shown in Figure 2.

The air entered the air return chamber from the inlet of the roof as shown in Figure 1. The main stream, which was heated by the right radiator, went through the right fan unit. After being blown into the tobacco curing chamber, the stream went through tobacco leaves, going up through the moisture discharged holes, the air holes and mostly the left fan unit. The air that went up through air holes and left fan unit returned the tobacco curing chamber through the left fan unit, and the air that went up through moisture discharged holes exited the barn with moisture.

### 2.2. Experimental Setup

**Figure 3:**
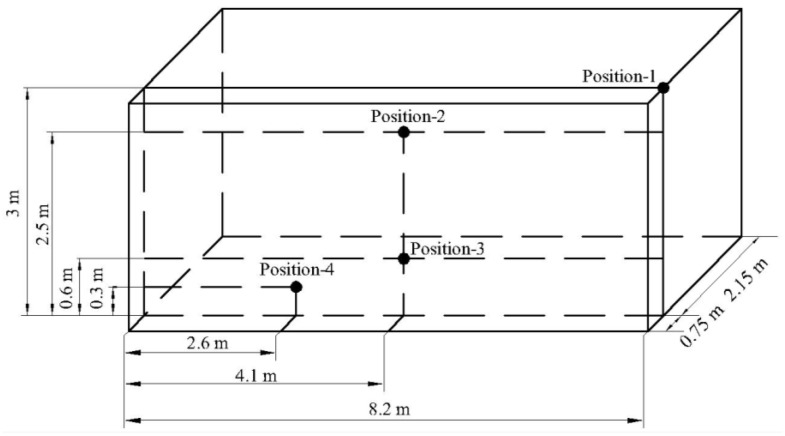
Distribution of temperature and humidity sensors.

**Figure 4:**
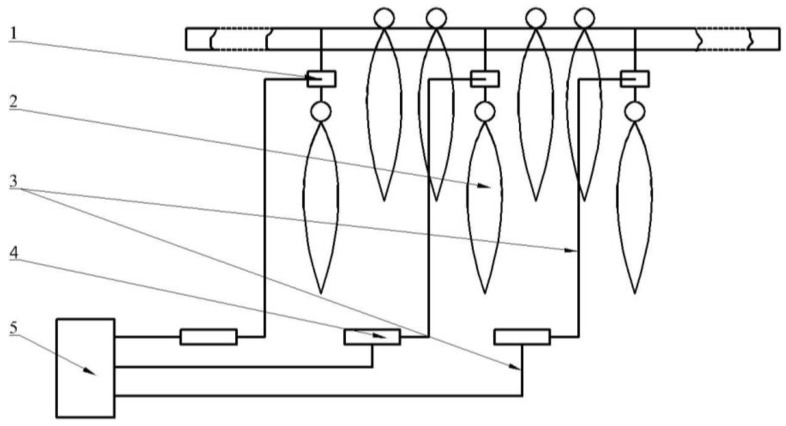
Schematic diagram of a gravity-measuring device. 1. Gravity sensors; 2. Tobacco leaves; 3. Signal lines; 4. Transmitters; 5. Paperless recorder.

The tobacco curing process was directed by a general purpose microcontroller, which controlled radiators and moisture discharged holes to satisfy the prescribed temperature and humidity. Four MIAOXIN TH10R-EX integrated temperature and humidity sensors (MIAOXIN, Zhejiang, China; temperature accuracy: 0.5 K; temperature range: 233 to 358 K; humidity accuracy: 5% RH; humidity range: 0 to 100% RH) consisting of sensor probes and data loggers were used to measure and record the temperature and relative humidity inside the tobacco curing chamber during tobacco curing process. Air temperature and relative humidity were logged at four positions for every 120 s as shown in Figure 3. One sensor probe (position-1) was mounted near to the right fan. Three sensor probes (position-2, position-3, and position-4) were fixed inside tobacco curing chamber at various locations. All the four positions were placed in the same vertical plane which was 0.75 m from the ahead vertical wall, and the data loggers connected with the four sensor probes were placed outside the barn. A gravity-measuring device consisting of MEACON MIK-LCS1 gravity sensors (MEACON, Zhejiang, China; accuracy: 0.03% FS; range: 0 to 10 kg), MEACON MIK-BSQW transmitters (MEACON, Zhejiang, China) and MEACON MIK-R200D paperless recorder (MEACON, Zhejiang, China) was applied to measure and record the tobacco gravity changes during tobacco curing process, which is shown in Figure 4. To capture the gravity changes inside the tobacco curing chamber, three gravity sensors were placed inside and weighed three sticks of tobacco leaves. Gravity signals transmitted on signal lines to transmitters, and were displayed and recorded by paperless recorder every 120 s. The air flow velocity was measured beside the right fan unit, using a TES-1340 hot-wire anemometer (TES, Taipei, Taiwan; accuracy: 0.01 m/s; range: 0 to 30 m/s).

### 2.3. Mathematical Modeling

The model of the tobacco curing process was based on the following assumptions: The process was modeled in 3D; The tobacco leaves zones were assumed to be porous medium; The evaporation of water in tobacco leaves was modeled, and the respiration of tobacco leaves during the curing process was neglected; The air flow in the tobacco leaves zones was regarded as incompressible and laminar due to the low velocity; The effect of the air return chamber and steel frame in the tobacco curing barn was neglected; And heat exchange with the surroundings was negligible.

The following governing equations depend on the assumptions mentioned above.

The continuity equation which can be also called the mass conservation equation for the fluid (air and water vapor) as follows [13]:

(1) $$\frac{\partial \rho }{\partial t}+\frac{\partial (\rho u)}{\partial x}+\frac{\partial (\rho v)}{\partial y}+\frac{\partial (\rho w)}{\partial z}=S_{M}$$

where *ρ* (kg·m^−3^) is the fluid density, *t* (s) is the time, *x*, *y*, and *z* (*m*) are the three space directions, *μ*, *ν*, and *ω* (m·s^−1^) are the velocity components according the direction of *x*, *y*, and *z*, and *S_M_* (kg·m^−3^·s^−1^) is the water vapor mass source.

The momentum equation as follows [13]:

(2) $$\frac{\partial (\rho u)}{\partial t}+div(\rho uu)=div(\mu grad\;u)-\frac{\partial p}{\partial x}+S_{u}$$

(3) $$\frac{\partial (\rho v)}{\partial t}+div(\rho vu)=div(\mu grad\;v)-\frac{\partial p}{\partial y}+S_{v}$$

(4) $$\frac{\partial (\rho w)}{\partial t}+div(\rho wu)=div(\mu grad\;w)-\frac{\partial p}{\partial z}+S_{w}$$

where *p* is the pressure (Pa), ***u*** is the velocity vector (m·s^−1^), μ is the dynamic viscosity of the fluid (kg·m^−1^·s^−1^), and *S_u_*, *S_v_*, and *S_w_* are the momentum source term components (N·m^−3^) according the direction of *x*, *y*, and *z*. The momentum source term contains viscosity loss and inertial loss for porous medium as follows [13]:

(5) $$S_{i}=\sum_{j=1}^{3}D_{ij}\mu v_{j}+\sum_{j=1}^{3}C_{ij}\frac{1}{2}\rho \left|v_{j}\right|v_{j}$$

where *i* = *u*, *v*, *w*, *D* and *C* are given matrix.

The energy equations as follows [13]:

(6) $$\frac{\partial [\gamma \rho_{f}E_{f}+(1-\gamma )\rho_{s}E_{s}]}{\partial t}+\nabla \cdot \left[v\left(\rho_{f}E_{f}+v\right)\right]=\nabla \cdot \left[K_{eff}\nabla T-\left(\sum_{i}h_{i}J_{i}\right)+\left(\tau \cdot v\right)\right]+S_{f}^{h}$$

(7) $$k_{eff}=\gamma k_{f}+(1-\gamma )k_{s}$$

where *E_f_* is the fluid energy (J·kg^−1^), and *Es* is the tobacco energy, *ρ_f_* is the tobacco density, *γ* is the porosity of the porous medium, T is the temperature (K), h_i_ is the enthalpy of species *i* (J·kg^−1^), *τ* is the shear stress tensor (Pa), and *S_f_^h^* is the heat source (W·m^−3^), *K_eff_* is the thermal conductivity (W·m^−1^·k^−1^) of the porous medium, *k_f_* is the thermal conductivity of the fluid, and *k_s_* is the thermal conductivity of the tobacco.

The transport equations for species s as follows [13]:

(8) $$\frac{\partial \left(\rho c_{s}\right)}{\partial t}+div\left(\rho uc_{s}\right)=div\left[D_{s}grad\left(\rho c_{s}\right)\right]+S_{s}$$

where *c_s_* is the volume fraction of species *s* (%), and *S_s_* is the production rate of water vapor (kg·m^−3^·t^−1^).

The Reynolds number in the tobacco curing chamber was calculated by using the following equation [14]:

(9) $$R_{e}=\rho vd\eta^{-1}$$

where *η* is the viscosity of the fluid (kg/ms). The κ-ε models remain popular because of their availability in user-friendly codes, which allow a straightforward implementation of the models, and because they are cheap in terms of computation time [15]. The standard κ-ε model has obtained applications in cheese-ripening room [16] and large food chillers room [17]. And the standard κ-ε model, proposed by Launder and Spalding in 1972, was used in this model [18].

### 2.4. CFD Grid

**Figure 5:**
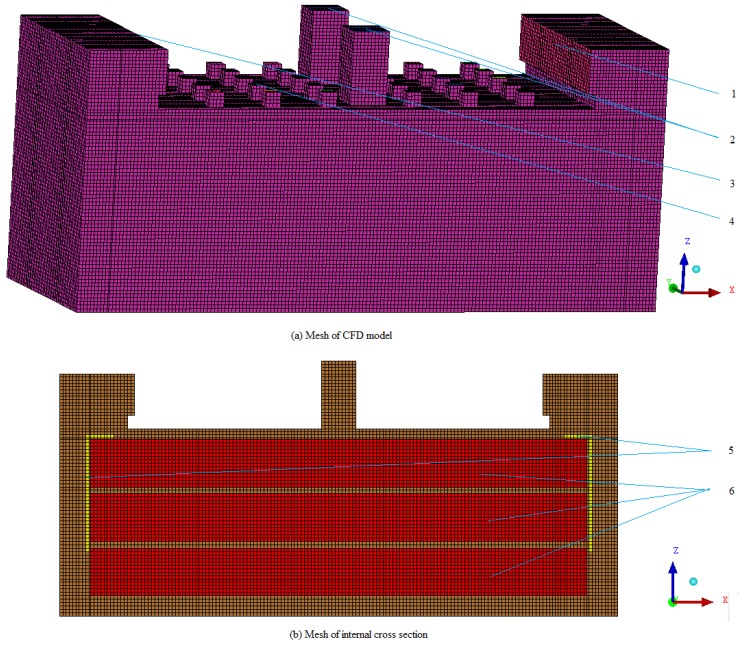
Computational fluid dynamics (CFD) mesh of the tobacco curing barn. 1. Velocity inlet; 2. Moisture discharged hole; 3. Outlet; 4. Air holes; 5. Bamboo curtain; 6. Porous zones.

The study focused on the tobacco curing chamber of the barn. Three-dimensional geometry of tobacco curing chamber was created, and the mesh was generated using ICEM CFD and exported to the ANSYS FLUENT for further processing. Figure 5 shows a schematic diagram of CFD mesh of the tobacco curing barn. Structured grid calculations usually take less time than an unstructured grid calculation [19]. A structured mesh made of hexahedral cells was used in this study. Blocking method is used for generating structured mesh, and mesh was equally spaced with 50 mm mesh spacing. Total numbers of faces are 1,944,245 with 635,102 cells. Minimum orthogonal quality was 1 and maximum aspect ratio was 3.67 which showed a good quality of the mesh.

### 2.5. Boundary Conditions

#### 2.5.1. Velocity Inlet

There was one velocity inlet, and the air velocity, which was measured by anemometer, had a uniform value of 3 m/s. The inlet air temperature and the mass fraction of water vapor were time dependent, and a user defined function (UDF) was used to describe the inlet air temperature and mass fraction of water vapor profiles with the time, on the basis of the measurement data of position-1 measured by temperature and humidity sensor. And turbulent intensity and hydraulic diameter was calculated by applying the following equations [20]:

(10) I=0.16Re−18

(11) $$D_{H}=4AP_{w}{}^{-1}$$

where *A* is the inlet area (m^2^), and *Pw* is the wetted perimeter (m).

#### 2.5.2. Pressure Outlet

There were thirty-three pressure outlets, thirty for the air holes, two for the moisture discharged hole, and one for the outlet (left fan unit). A pressure of 1 atm was assumed at all the pressure outlet. Turbulent intensity and hydraulic diameter was calculated by using Equations (10) and (11).

#### 2.5.3. Walls

All the boundaries except the velocity inlet and pressure outlets boundaries were the walls, and all the walls were assumed to be insulated. The standard wall functions were applied on the walls.

#### 2.5.4. Porous Medium

**Figure 6:**
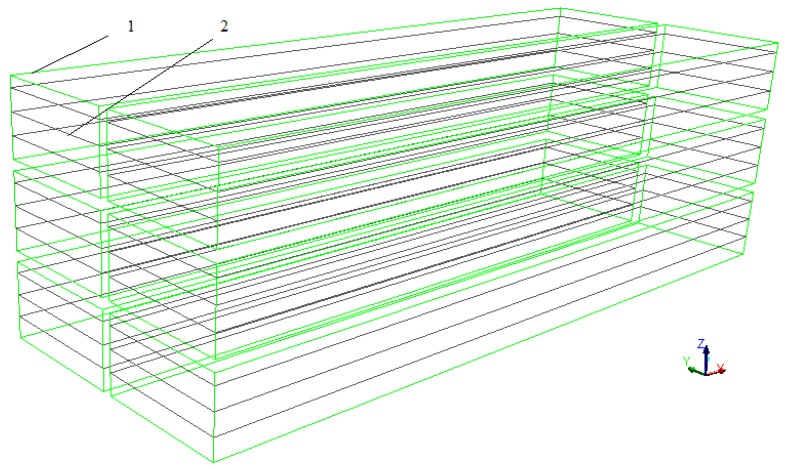
Schematic diagram of eighteen surfaces. 1. Porous zones; 2. Surfaces.

The tobacco leaves were placed closely enough and simplified to six porous zones, with three columns and two rows. Each of the six porous zones (the green area) was 7.3 m long, 1.3 m wide and 0.7 m high as shown in Figure 6. The porosity of porous zone was calculated by applying the following equations [20]:

(12) $$r=1-\frac{\rho_{1}}{\rho_{d}}$$

where *ρ*_1_ = 45.5 kg/m^3^ is the bulk density, and *ρ_d_* = 515 kg/m^3^ is the apparent density [21]. Viscous resistance factor and inertial resistance factor were used to calculate the momentum source term, and they were calculated by applying the following equations [20]:

(13) $$\frac{1}{\alpha }=\frac{150（1-r{）}^{2}}{{d_{p}}^{2}r^{3}}$$

(14) $$C_{2}=\frac{3.5（1-r）}{d_{p}r^{3}}$$

where *d_p_* = 0.01 m is the particle average diameter, and r is the porosity of porous zone. The particle average diameter was regarded as thickness of tobacco leaves in this model.

#### 2.5.5. Discrete Phase Model

In order to introduce water droplets into the curing barn from tobacco leaves during the tobacco curing process, the discrete phase model (DPM) and species transport model in ANSYS FLUENT were implemented. The water droplets were injected into the tobacco curing chamber from eighteen surfaces (the black planes), which distributed throughout the porous zones (the green areas) as shown in Figure 6. The surfaces injected 148,176 particle parcels per DPM iteration. The total mass flow of water droplets was calculated from the gravity changes of tobacco leaves measured by gravity sensors. According to the measurement data of gravity changes, the whole tobacco curing process can be divided into four phases, and the total mass flow of water droplets were 0.00465 kg/s from 0 to 57,600 s, 0.00295 kg/s from 57,600 to 141,000 s, 0.0036 kg/s from 141,000 to 344,400 s and 0 kg/s from 344,400 to 397,200 s, respectively. This model can realize the water droplets evaporating inside the porous zones just like the actual situation in which water evaporate inside tobacco.

#### 2.5.6. Initial Conditions

Prior to the experiment, sticks of tobacco leaves were placed on the steel frame in the tobacco curing barn. The initial temperature and relative humidity of the barn was assumed to be uniform at 303 K and 100% RH, which was measured by temperature and humidity sensors. The simulation used a transient, pressure-based solver and SIMPLE pressure–velocity coupling algorithm. And the time step size was chosen to be 120 s, which had the same interval as the data acquisition sensors in the experiment.

## 3. Results and Discussion

### 3.1. Grid Sensitivity and Computational Validation

**Figure 7:**
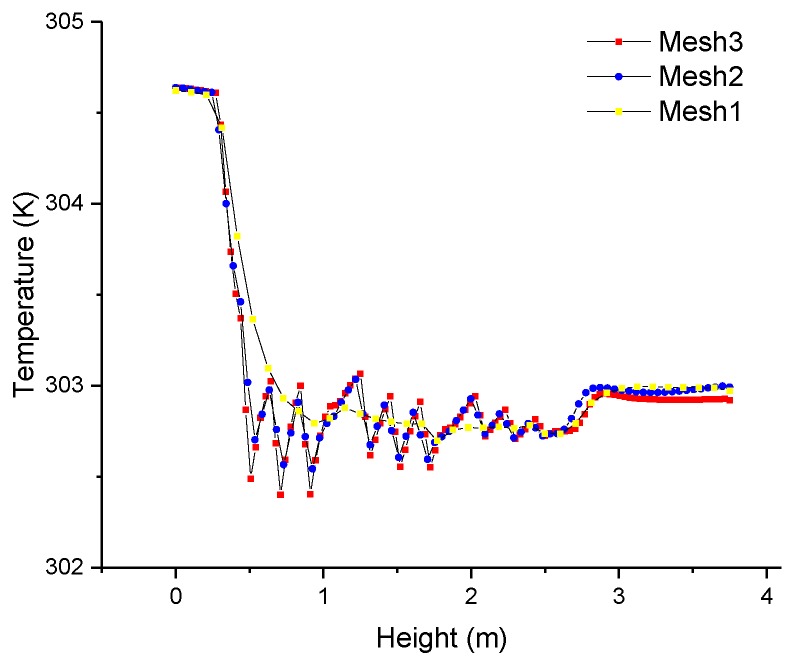
Temperature profiles along the vertical line for mesh1, mesh2 and mesh3.

Grid sensitivity tests were performed employing the three different meshes summarized in Table 1. A vertical line was in the middle of a vertical plane, which was 0.75 m from the ahead vertical wall. The temperature profiles along the vertical line for the three meshes were compared in Figure 7. Mesh1 (the coarsest) did not show the same temperature profile as observed with mesh2 and mesh3. Mesh3 (the finest) can result in very high resolution of computational results, but it needed more time for calculation. It was concluded that the optimal computational mesh was mesh2.

**Figure 8:**
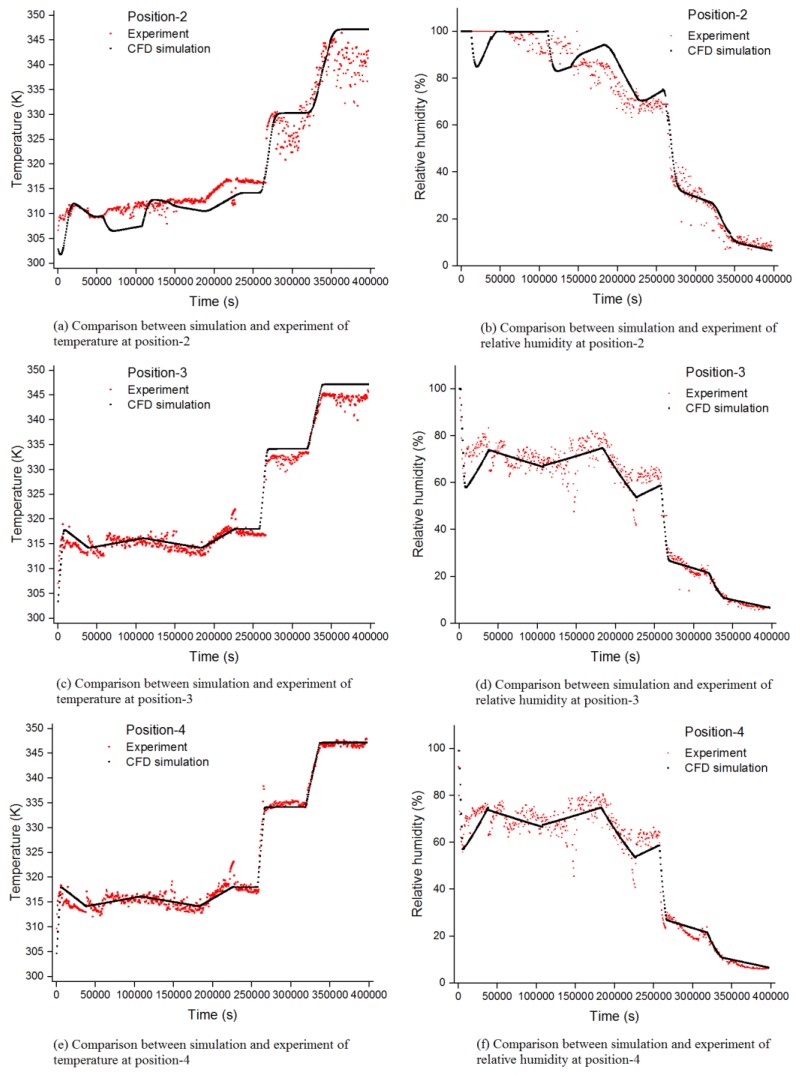
Comparison between CFD simulation and experiment of the temperature and relative humidity at position-2, position-3 and position-4.

Three-dimensional transient state computations have been performed. The time wise temperature and humidity profiles in the tobacco curing barn were measured by the four integrated temperature and humidity sensors. The experimental temperature and humidity measurements at position-1 were used for the inlet boundary condition. In order to validate the CFD simulation model for the tobacco curing barn, the temperature and relative humidity predictions at position-2, position-3 and position-4 were compared with the experimental temperature and relative humidity measurements in Figure 8. The temperature and relative humidity predictions showed good agreement with the experimental temperature and humidity measurements. In Figure 8a,b, the difference between predictions and experimental measurements at the beginning of the curing process at position-2 can be explained by the fact that in the experiment, the humidity at position-2 was higher than we measured. The errors existed in the measurements, which were measured by integrated temperature and humidity sensors and gravity sensors. The gravity measurements were used to estimate the gravity of tobacco leaves in the tobacco curing barn. There were also some discrepancies between predictions and experimental measurements at position-2 and position-3 in Figure 8a–d, and they were mainly caused by the water estimation error in tobacco leaves.

As presented in Table 2, comparing the simulated temperature and relative humidity profile to the measured data at position-2, position-3 and position-4, leads to an average RMS error of 2.61% for temperature and 5.66% for relative humidity, while the average coefficient of correlation between the simulated values and the measured values were 0.98 and 0.98 for temperature and relative humidity, respectively. Because of the high coefficient of correlation and low RMS error, the predicted temperature and humidity profiles can be used to estimate and analyze the temperature and humidity distributions in the tobacco curing barn.

### 3.2. Temperature Distributions

According to the tobacco curing process, it can be divided into four stages, 1 s to 210,000 s, 210,001 s to 255,000 s, 255,001 s to 315,000 s, 315,000 s to 397,200 s. Each stage can be divided into temperature rise and temperature control, and the temperature control takes much longer than the temperature rise.

**Figure 9:**
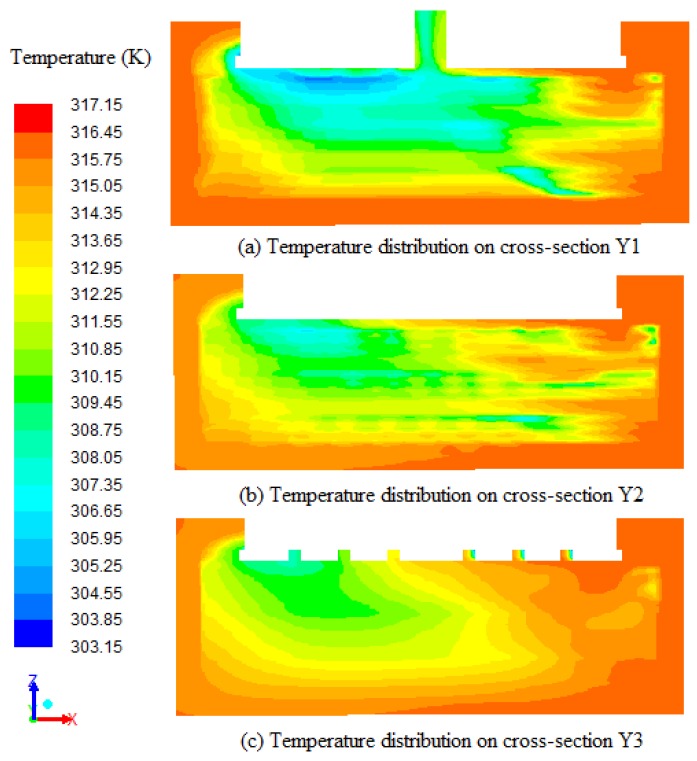
Temperature distributions comparing three different cross-sections (Y1, Y2 and Y3) of the tobacco curing chamber at 108,000 s.

The temperature distributions comparing three different cross-sections of the tobacco curing chamber at 108,000 s are presented in Figure 9. The cross-section Y1 (0.75 m from the ahead vertical wall) cross the middle of three left porous zones. And the cross-sections Y2 (1.35 m from the ahead vertical wall) and cross-sections Y3 (1.45 m from the ahead vertical wall) cross the edge of three left porous zones and the middle of the tobacco curing chamber, respectively. It can be observed from Figure 9 that the temperature of bottom region is higher than the top region, and the temperature of inlet region is higher compared to the outlet region. The temperature distributions on cross-section Y1 are more uniform compared to the cross-section Y2 and Y3. And the direction of airflow is from right to left. The average temperatures on cross-section Y1, cross-section Y2 and cross-section Y3 are 312.55 K, 313.12 K and 313.82 K, respectively. This demonstrates that the inside of the porous zones stays about 1 K cooler than the outside temperature; this could be the reason of water evaporating and heat absorption inside the porous zones.

**Figure 10:**
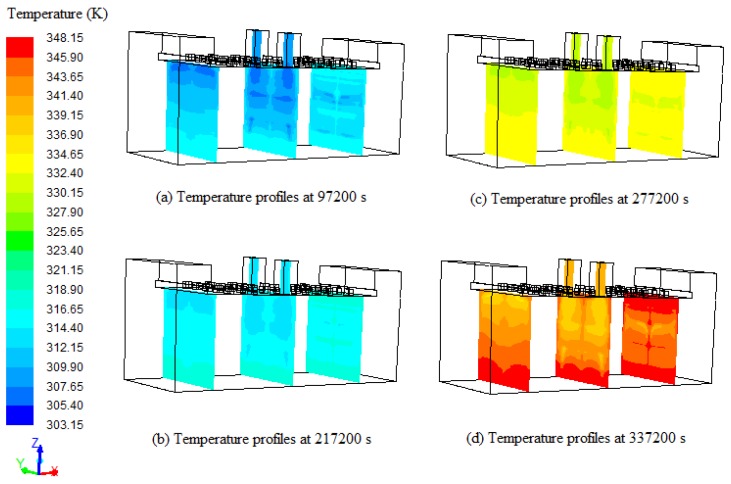
Temperature profiles across three different cross-sections (1.3 m, 4.2 m, and 6.7 m from the left wall) at (**a**) 97,200 s; (**b**) 217,200 s; (**c**) 277,200 s and (**d**) 337,200 s.

The temperature profiles across three different cross-sections at 97,200 s, 217,200 s, 277,200 s and 337,200 s of tobacco curing time are presented in Figure 10. The left cross-section is 1.3 m from the left wall, the middle cross-section is 4.2 m from the left wall, and the right cross-section is 6.7 m from the left wall. It can be seen from the Figure 10 that the temperatures of porous zones increase with the increase of temperature in the curing barn, and the average temperature differentials on the left cross-section, middle cross-section and right cross-section are 6 K, 4.5 K and 1.5 K, respectively. The temperature differential on the left cross-section is 4.5 K more compared to the right cross-section, which may be due to the direction of airflow. The temperatures in the top region of the tobacco curing barn are slightly less compared to the bottom region, and the low temperature regions on the top of middle cross-section are larger compared to the left cross-section and right cross-section, which may be due to the cool air accumulation at the top. Also, the temperature gradients on the left cross-section are obvious, with low temperatures on the top and high temperatures on the bottom. It can be observed from Figure 10 that the temperature distributions in the tobacco curing barn are similar in both sides of the porous zones and in the four stages of the tobacco curing process. According to the simulated temperature profiles, the temperature distributions in the tobacco curing barn can be predicted using the measurements taken by temperature sensors.

### 3.3. Water Vapor Mass Fraction Distributions

**Figure 11:**
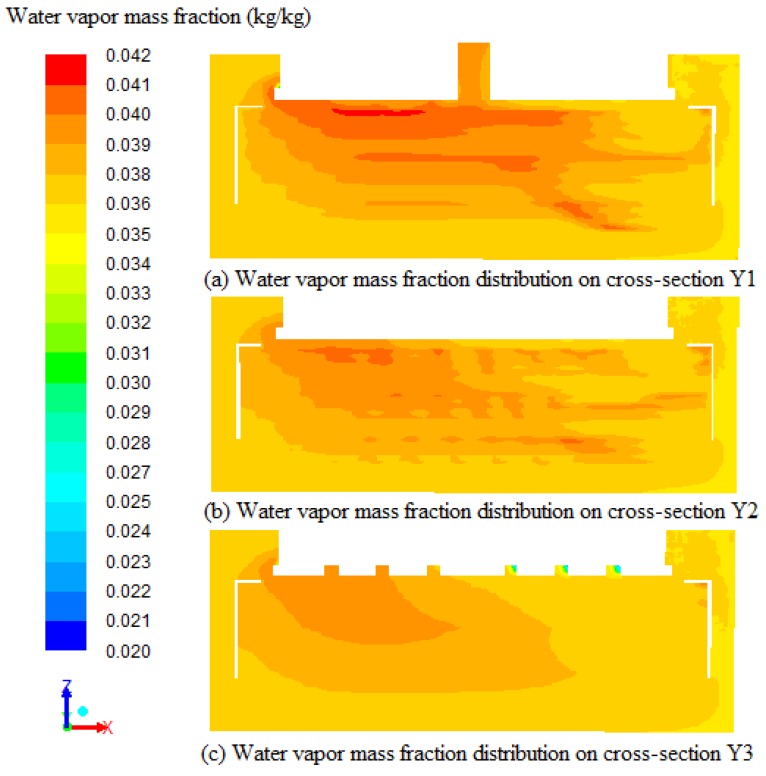
Water vapor mass fraction distributions on the cross-sections (Y1, Y2, and Y3) of the tobacco curing chamber at 108,000 s.

The water vapor mass fraction distributions on the cross-section Y1, cross-section Y2 and cross-section Y3 at 108,000 s are presented in Figure 11. It can be seen from Figure 11 that the mass fraction of vapor near the top wall is more when compared to the center and bottom of the tobacco curing barn due to the low density of vapor and the direction of air flow. The temperature gradients on the left side are more obvious compared to the right side on the cross-section Y1, cross-section Y2 and cross-section Y3 with high vapor mass fraction on the top and low vapor mass fraction on the bottom. The average water vapor mass fractions on cross-section Y1, cross-section Y2 and cross-section Y3 are 0.0371 kg/kg, 0.0369 kg/kg and 0.0366 kg/kg, respectively. This demonstrates that the inside of the porous zones stays about 0.0005 kg/kg more than the outside water vapor mass fraction, and this also can be the result of water evaporating inside the porous zones. Water vapor accumulations form the high vapor mass fraction regions on the top of the tobacco curing barn. The high vapor mass fraction regions have low temperature because of the water evaporating. The same result can be obtained in Figure 9. This means that the water vapor mass fraction distributions are related with the temperature distributions on the same position.

### 3.4. Porosity and Particle Average Diameter

**Figure 12:**
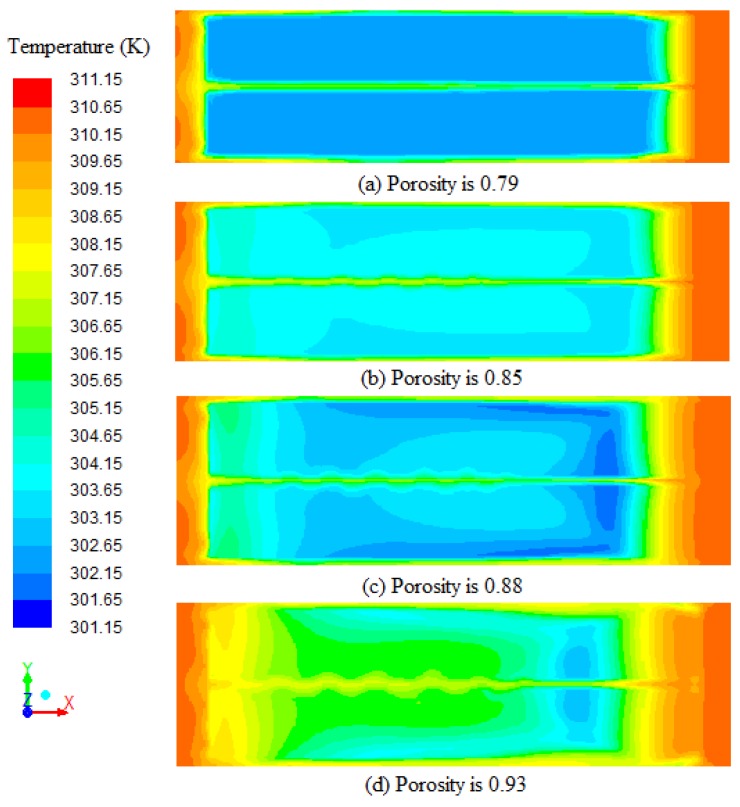
Temperature profiles on the cross-section (0.8 m from ground) at 3000 s with different porosities (a = 0.79, b = 0.85, c = 0.88, and d = 0.93) of porous zones.

The porosity can be calculated with Equation (12), and tobacco leaves with higher porosity had lower loading density. Figure 12 shows the temperature profiles on the cross-section (0.8 m from ground) at 3000 s with different porosities of porous zones. The average temperatures on the cross-section with different porosities (a = 0.79, b = 0.85, c = 0.88, and d = 0.93) are 303.92 K, 305.22 K, 304.98 K and 306.78 K, respectively. The average temperature differential between Figure 12a,d can be 2.86 K under the same circumstance. The average temperature increases with the increase of porosity. The average temperature in Figure 12b is slightly higher compared to Figure 12c, because the air which heat is absorbed by water evaporation moves more slowly. The temperature profiles in Figure 12 clearly emphasize that increase in porosity causes an enhanced heat transfer. This shows that with higher tobacco loading density, there is a negative effect on heat transfer.

**Figure 13:**
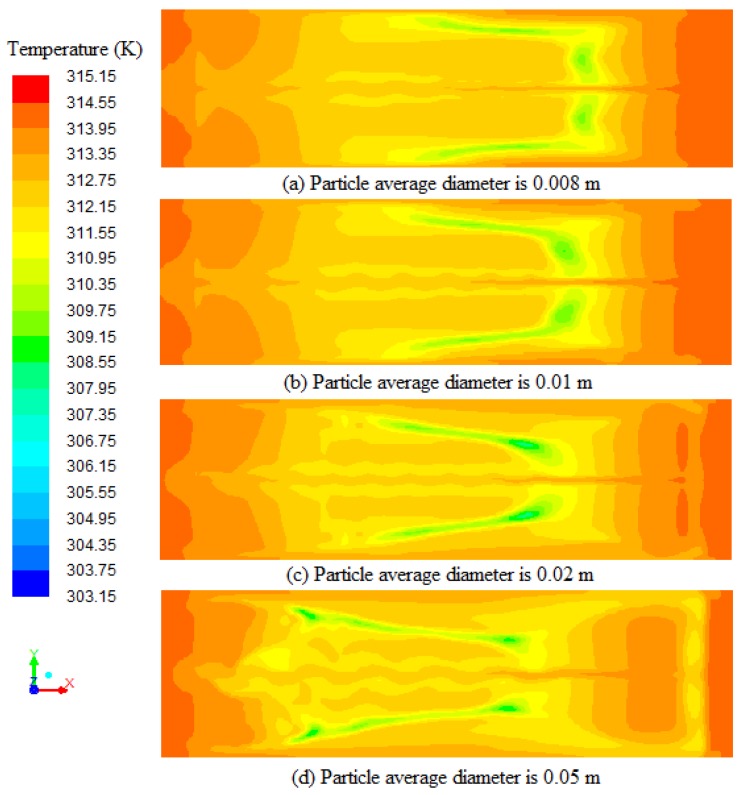
Temperature profiles on the cross-section (0.8 m from ground) at 38,400 s with different particles average diameters (a = 0.008 m, b = 0.01 m, c = 0.02 m, and d = 0.05 m) of porous zones.

The particle average diameter had a relationship with the flow resistivity and the bulk density, affecting the movement of airflow [22]. In this model, the particle average diameter is relative to thickness of tobacco leaves, and different thicknesses of tobacco leaves are assumed to have the same capacity of moisture retention. Figure 13 depicts the temperature profiles on the cross-section (0.8 m from ground) at 38,400 s with different particles average diameters (a = 0.008 m, b = 0.01 m, c = 0.02 m, and d = 0.05 m) of porous zones. It can be seen from the Figure 13 that the low temperature region (the green area in Figure 13) appears at 1.9 m, 2.1 m, 2.4 m and 2.9 m from the right wall with the increase of the particle average diameter, and the direction of airflow is from right to left. The low temperature region means position of the air which heat is absorbed by water evaporation. The air in Figure 13d moves faster than the air in Figure 13a. This shows that the heat transfer become faster with the increase of thickness of tobacco leaves.

## 4. Conclusions

A 3D transient CFD model was developed for the new bulk tobacco curing barn using porous medium, species transport, κ–ε turbulence and discrete phase models in the present study. This model can realize the water droplets evaporating inside the porous zones just like the actual situation where the water evaporates inside tobacco. The following conclusions will be helpful to optimize the process of tobacco curing.

(1) The CFD model was validated using the temperature and relative humidity data that were recorded during the tobacco curing process using integrated temperature and humidity sensors. Comparison of simulation results with experimental data showed a fairly close agreement. The CFD model is able to produce a good qualitative insight into the temperature and humidity distribution in the new bulk tobacco curing barn.

(2) The temperature of the bottom region was higher than the top region in the barn. The temperature decreased following the direction of airflow, and the temperatures inside porous zones were higher than outside porous zones. According to the temperature distribution in tobacco curing chamber, thick leaves which need higher temperature can load on the bottom and inlet area, and thin leaves which need lower temperature can load on the top and outlet area. Water vapor concentrated on the top and outlet area in the barn, and the position of moisture discharged holes can be adjusted due to the distribution of water vapor.

(3) Tobacco loading density and thickness of tobacco leaves had an explicit effect on the temperature distributions in the barn. Tobacco loading density had negative effect on heat transfer, and thickness of tobacco leaves had a positive effect on heat transfer. The curing technology can be adjusted due to the actual tobacco loading density and thickness of tobacco leaves.

The temperature and humidity distributions in the tobacco curing barn can be studied by using the CFD model. Additionally, during the curing process, the temperature and humidity distributions can be predicted by the measurements measured by limited temperature and humidity sensors. This can be used for tobacco curing process and barn design optimization, and it can save the cost and improve the tobacco curing quality and production efficiency.

## Figures and Tables

**Table 1:** Information on the meshes in the sensitivity tests.

	Mesh1	Mesh2	Mesh3
Cells	65,355	635,102	1,973,366
Faces	204,666	1,944,245	6,003,052
Nodes	741,88	674,531	3,057,028

**Table 2:** Root mean square (RMS) error and coefficient of correlation compared to the measured temperature and relative humidity.

	P-2 (T/K)	P-2 (RH/%)	P-3 (T/K)	P-3 (RH/%)	P-4 (T/K)	P-4 (RH/%)
Coefficient of correlation	0.97	0.98	0.99	0.98	0.99	0.98
RMS error	3.88	6.57	2.61	5.27	1.34	5.13
